# The Human Mucosal Mycobiome and Fungal Community Interactions

**DOI:** 10.3390/jof3040056

**Published:** 2017-10-07

**Authors:** Elizabeth A. Witherden, Saeed Shoaie, Rebecca A. Hall, David L. Moyes

**Affiliations:** 1 Centre for Host-Microbiome Interactions, Dental Institute, King’s College London, London SE1 9RT, UK; elizabeth.witherden@kcl.ac.uk (E.A.W.); saeed.shoaie@kcl.ac.uk (S.S.); 2 Centre for Translational Microbiome Research, Department of Microbiology, Tumor and Cell Biology, Karolinska Institute, SE-171 77 Stockholm, Sweden; 3 Institute of Microbiology and Infection, School of Biosciences, University of Birmingham, Birmingham B15 2TT, UK; r.a.hall@bham.ac.uk

**Keywords:** mycobiome, microbiome, bacterial-fungal interactions, fungal-fungal interactions, host-fungal interactions, systems biology

## Abstract

With the advent of high-throughput sequencing techniques, the astonishing extent and complexity of the microbial communities that reside within and upon us has begun to become clear. Moreover, with advances in computing and modelling methods, we are now beginning to grasp just how dynamic our interactions with these communities are. The diversity of both these communities and their interactions—both within the community and with us—are dependent on a multitude of factors, both microbial- and host-mediated. Importantly, it is becoming clear that shifts in the makeup of these communities, or their responses, are linked to different disease states. Although much of the work to define these interactions and links has been investigating bacterial communities, recently there has been significant growth in the body of knowledge, indicating that shifts in the host fungal communities (mycobiome) are also intimately linked to disease status. In this review, we will explore these associations, along with the interactions between fungal communities and their human and microbial habitat, and discuss the future applications of systems biology in determining their role in disease status.

## 1. Introduction

With the global burden of fungal diseases rising, researchers have begun to turn to next-generation sequencing (NGS) technology to investigate the role fungi play in the spectrum of human health and disease. At the forefront of this advancement is the “Superorganism” hypothesis, where humans are considered to be complex organisms made up of numerous mutually independent smaller organisms (i.e., bacteria, fungi, virus, archaea) and their genomes. This group of microbial cells and their genomes are collectively referred to as the human microbiota and microbiome, respectively. Over the past decade, the bacterial portion of the microbiome has been well characterized in a number of health and disease states of man, including: Type 2 diabetes [1,2,3]; liver cirrhosis [4]; colon cancer [5]; rheumatoid arthritis [6], and; inflammatory bowel disease [7,8,9]. In contrast, however, research into the mycobiome (the fungal proportion of the microbiome) has received less attention, such that the field of mycobiome research is still in its infancy. 

There are currently several common challenges facing microbiome and mycobiome researchers. First, irrespective of their biomass, fungi account for a relatively small percentage of the human microbiome compared to their bacterial counterparts [10,11]. Second, similar to what we have seen with bacteria, the isolation of nucleic acids from fungal cells can be problematic, and often requires a combination of enzymatic, chemical and mechanical lysis steps [12]. Third, the ability to discriminate between fungal taxa is influenced by sequencing primer choice and, finally, curated databases for taxonomic assignment and/or the annotation of fungal genomes are lacking or are incomplete [13,14]. It is against this backdrop that a number of authors have begun to unravel the mystery of the human mycobiome. 

Akin to the microbiome, the human mycobiome has been shown to play an integral role in the pathology of health and disease in man [15]. In fact, changes to the mycobiome have been shown to play vital roles in the modulation of the host immune response [16,17], disease progression [16], the maintenance of microbial population structures [18], as well as metabolic functioning of the host [18]. 

This review aims to explore the current status of human mucosal mycobiome research, focusing on the gastrointestinal tract.

## 2. The Mycobiome

### 2.1. Studying the Mycobiome

The advancements we have seen in high-throughput NGS technology over the past decade, has dramatically changed the landscape against which we study the mycobiome. From traditional, culture-based methodologies, we have moved towards the use of amplicon based technologies that target fungal specific house-keeping genes, which allow researchers to identify both cultivatable and non-cultivatable fungal species in a wealth of environmental samples. Theses fungal house-keeping genes are situated within the fungal ribosomal RNA gene cluster (rRNA), and include the 18S rRNA, 5.8S rRNA and 28S rRNA genes, as well as the internal transcribed spacer regions (ITS1 and ITS2) [19]. Similar to what has been seen with the 16S rRNA gene in amplicon-based bacterial microbiome studies [20], there is currently a lack of consensus between authors regarding which genetic target offers the best level of taxonomical and phylogenetic resolution [13], and as such several alternative primer sets exists that target different regions of these fungal genes (Cui et al. [19] gives a good overview of the different fungal rDNA primers used in mycobiome studies to date). Confounding this issue in mycobiome studies is the lack of completely sequenced and annotated fungal genomes that can be used for taxonomic identification. Current fungal rRNA databases routinely used to assign fungal taxonomy in microbiome studies include UNITE [21] for ITS, SILVA [22] for fungal 18S and 28S rRNA genes, as well as RDP [23] for fungal 28S rRNA genes. 

Unlike the field of microbiome research, mycobiome studies tend not to use shotgun metagenomic sequencing approaches. As metagenomic approaches simultaneous sequence all of the genetic material within a sample (host, bacterial, fungal, archael, etc.), they have the potential to generate both taxaonomic and functional information. However, this technique relies on a lot of computational power and is limited by the inclusion of both bacterial, fungal and archael genes in reference catalogs [24]. In fact, in a current metagenomic reference catalog used for studying gut microbial populations [24,25], only 0.1% of the 3.3 million reference genes were reported to be of eukaryotic origin [24]. Until we overcome the limitation posed by a lack of fungal reference genes in these catalogs, the true potential of mycobiome research using metagenomic approaches cannot be fully realized.

### 2.2. Mucosal Mycobiomes in Health and Disease

**Figure 1:**
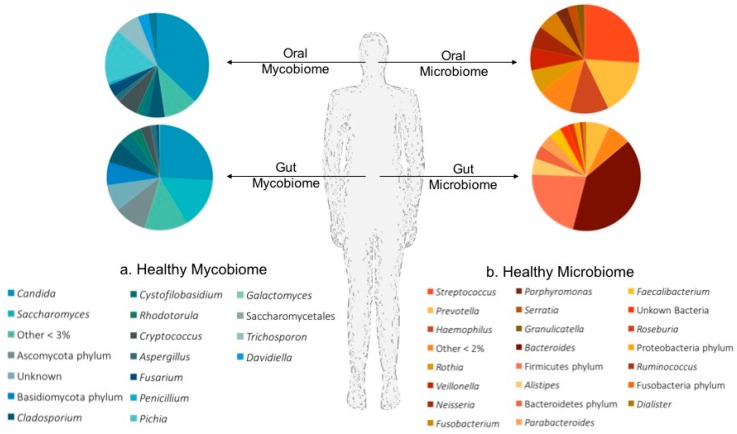
Healthy microbial communities of the human body. An overview of the Mycobiota (**a**) and Microbiota (**b**) identified on the mucosal surfaces of the oral cavity and lower gastrointestinal tract in a state of health. Pie charts depict the average relative abundance of fungal taxa (**a**); and bacterial taxa (**b**) observed in the healthy oral cavity [26], and gastrointestinal tract [27] of study participants.

There is mounting evidence linking the host’s mucosal microbiomes to the modulation of host immunity. One’s ability to untangle the complex interactions between the microbiota, mycobiota and immune response at a given body site begins with developing an understanding of which microbes frequently call these mucosal niches home. A summary of our current knowledge of the mycobiota and microbiota that colonize the oral cavity and the lower gastrointestinal tract (GIT) in states of health is given in Figure 1.

#### 2.2.1. The Oral Mycobiome

The concept of a “core healthy oral mycobiome” was introduced in 2010 by Ghannoum and colleagues when they characterized the oral mycobiome of 20 healthy adults [28]. In this study interrogation of ITS1F/ITS2 sequences identified a total of 85 fungal genera within the oral cavity, 11 of which related to non-culturable fungal genera [28]. Although the exact number of fungal genera in the oral cavity varied between participants (range 5–39), a core set of genera were identified in the oral cavities of more than 20 percent of study participants: *Candida* (75%); *Cladiosporium* (60%); *Aureobasidium* (50%); *Aspergillus* (35%); *Fusarim* (30%), and; *Cryptococcus* (20%). The high prevalence of *Candida* in the oral cavity is consistent with previous culture-based studies, and subsequent molecular studies confirmed the high prevalence of *Candida* spp. within the oral cavity, reporting *Candida albicans*, *Candida parapsilosis* and *Candida dubliniensis* as the most abundant oral Candida species [26,29,30].

The constituents of the “core healthy oral mycobiome” were refined in 2014, when Dupuy and colleagues identified only eight of the key oral mycobiome genera originally classified by Ghannoum et al. in their healthy saliva samples [29]. This highlights that although a healthy core oral mycobiome may exist, the overall abundance and diversity of fungal taxa may be somewhat individualised. One of the most interesting aspects of this study was the report of a relative high abundance (13–96%) of *Malassezia* within the oral cavity of their entire study cohort, which is in contrast to previous studies which failed to identify *Malassezia* spp. at all [28,29]. Although, subsequent molecular studies are yet to confirm the reports of *Malassezia* within the oral cavity of man, its presence can be logically explained. First, *Malassezia* is a common skin commensal that has been isolated from the nares [31] and respiratory tract [32] of man, thus its presence in the oral cavity is not unexpected. Secondly, as *Malassezia* has a relatively robust cell wall structure, the choice of cell lysis methodology may significantly affect the ability to isolate *Malassezia* DNA, resulting in a subsequent underestimation of fungal abundance [12,29]. In light of this, it is important to consider here the differences in the DNA extraction processes used in the two studies. In fact, both studies used the same FAST DNA Spin Kit for DNA isolation, however, Dupuy et al. modified the protocol to include a robust mix of ceramic and zirconia beads to facilitate mechanical digestion, and also tripled the timing at the homogenization step [29].

The importance of bacterial-fungal, and fungal-fungal interactions in the homeostatsis of oral health, was recently highlighted in individuals with and without HIV [26]. In this study, the authors concurrently profiled the microbiome and mycobiome in the oral cavity (Figure 1) of 24 subjects and identify a number of significant fungal-fungal correlations in individuals with and without HIV. Although both *Candida* and *Penicillium* were isolated from the oral cavity of all individuals, significant differences in the overall mycobiome profiles were identified between the health and disease states [26]. For example, *Alternia*, *Epicoccum* and *Trichosporon* were only found in HIV positive patients, whilst *Pichia*, *Cladosporium* and *Fusarium* were associated with health. In contrast, assessments of the microbial populations, identified a stable oral microbiome between the two groups, predominated by *Streptococcus* and *Prevotella.* When the authors evaluated the bacterial-fungal relationships in this dataset, they identified a number of significant correlations, including a significant negative correlation between the abundance of *Rothia* and *Cladosporium* in the oral cavity of healthy individuals, although no mechanistic justification for this correlation has been given. Interestingly, the authors go on to identify an antagonistic effect between the oral fungal genera *Candidia* and *Pichia*, such that a relative increase in Pichia colonisation was associated with a reduction in the abundance of *Candida* [26]. Highlighting the importance for elucidating the role of bacterial-fungal and fungal-fungal interactions on microbiome and mycobiome community structures as well as health and disease. 

#### 2.2.2. The Gut Mycobiome

Perhaps the most widely studied fungal niche in humans is the gastrointestinal tract. The higher burden of fungal cells in the gut compared to other body niches, along with the wealth of data linking the gut microbiome to systemic inflammation makes the gut mycobiome an important area of study. Numerous authors have begun to unravel the role of the mycobiome in gut health [27], and disease, including; inflammatory bowel disease (IBD) [8,9,33], obesity [34], and inflammation [16,17,35].

Molecular studies of the gut mycobiome, have identified that healthy stools contain fungal genera belonging predominately to either the Ascomycota or Basidiomycota fungal taxa [27,33]. Furthermore, these studies report a rich and diverse fungal community within the GIT of healthy individuals which is predominated by *Candida*, *Saccharomyces*, *Trichosporon* and *Cladosporium* [16,27,36]. 

In 2016, Mar Rodriguez et al. [34], investigated the role of the gut mycobiome in obesity, and showed that although there was no significant difference in mycobiome richness between obese and non-obese individuals, the specific composition of the mycobiome could distinguish between obese and non-obese individuals. In this respect, the obese mycobiome, was predominated by *Candida*, *Nakaeseomyces*, *Penicillium* and *Pichia*, whilst in the non-obese mycobiome *Mucor*, *Candida* and *Penicillium* were the most prevalent. Interestingly, the authors showed that the genus *Mucor* correlated negatively with metabolic markers of obesity (fasting triglycerides, low-density lipoprotein (LDL), cholesterol, BodyMass Index (BMI) and fat mass), whilst the genus *Penicillium* and the family *Aspergillaceae* correlated positively with high-density lipoprotein (HDL) [34]. Although, this study did not elucidate the role of the diet in obesity and mycobiome composition, a previous study by Hoffmann et al. [27] uncovered interactions between diet and the gut mycobiome. In that study Hoffmann et al. concurrently profiled the mycobiome and microbiome in stools (Figure 1), and showed that a healthy gut mycobiome is predominated by the fungal genera *Candida* and *Saccharomyces*, and the gut microbiome by Bacteroidetes and Firmicutes taxa. Of particular interest here were the positive associations of *Candida* with a carbohydrate-rich diet, and its co-occurrence with particular bacterial (*Prevotella* and *Rumminococcus*) and archaeal genera (*Methanobrevibacter*). 

Taken together, this data provides strong support for the role of bacterial-bacterial and fungal-bacterial interactions in host metabolism, and systemic inflammatory conditions. 

A number of studies have begun to untangle the relationship between intestinal mycobiota and IBD [8,9,33]. In these studies, the gut mycobiome of patients with IBD is characterized by a reduction in fungal biodiversity and a change in community composition compared to healthy controls. More specifically, at the phyla level, the ratio of fungi belonging to the Basidiomycota and Ascomycota taxa has been shown to be altered when compared to healthy controls, such that there is a statistically significant increase in Basidiomycota taxa at the expenses of Ascomycota seen in IBD [8]. The hallmarks of this dysbiosis appear to develop from a reduction in the relative abundance of *Saccharomyces*, *Penicillium* and *Kluyveromyces* coupled with an increase in *Candida*, *Malasseziales* and *Filobasidiaceae* in IBD compared to healthy controls [8]. Interestingly although both these studies reported a decrease in *Saccharomyces cerevisiae* and increase in *Candida* in stools from IBD cases, the exact species of *Candida* differed between the studies: Sokol reported an increase in *C. albicans* [8], whilst Hoarau reported an increase in *Candida tropicalis* [9]. These differences might be explained by the different extraction methodologies used, but this variation is most likely explained by the different ITS sequencing targets used in the two studies. Where the Sokol study targeted ITS2 and the Hoarau study targeted ITS1 [8,9].

## 3. Fungal Interactions

When thinking about the role that a specific species, or the fungal community as a whole, may play in host health and disease, it is important to remember that these species and communities do not exist in isolation. Recent developments suggest that although the microbiome and mycobiome impact on the host they also affect each other, and the host will also impact on the homeostasis of these species through the production of metabolites and other, more specific factors and interactions. 

### 3.1. Polymicrobial Interactions and the Microbiome

Although there are a significant number of studies exploring the bacterial communities, the importance of communities of fungal species in regulating the composition of the microbiome as a whole is only now beginning to be explored. In particular, the role of metabolites in these interactions is finally beginning to be explored [37]. The microbiota forms a complex ecosystem of cooperating microbes. Within this ecosystem each species will be producing metabolic intermediates, signalling molecules and toxins that will accumulate and impact on the physiology of other members of the community. Metabolic approaches confirm that growth within a polymicrobial community results in alterations to the global metabolome that are dependent on the species present in the community. These polymicrobial communities also result in the production of new secondary metabolites as a result of the action of multiple species in a chain of events, which may offer clinical significance. For example, mixed communities of *Cladosporium* and *B. subtillis* resulted in the production of a novel secondary metabolite that displayed antimicrobial properties, as well as an increase in surfactins [38]. Co-culturing *Rhizopus* microspores with *Burkholderia gladioli* also induces the production of bongkrekic acid—a notable respiratory toxin [39]. Therefore, although in their infancy, these types of studies provide evidence that polymicrobial communities (like those found in the microbiome) affect secondary metabolite production, which may affect the community structure as well as its interactions with the host habitat. For example, the presence of *C. albicans* in oral biofilms promotes the growth of *S. mutans* through the induction of genes involved in metabolic pathways [40]. The close-knit community of the microbiome, in conjunction with alterations in metabolic flux, will also set up micro-domains of differing environmental parameters (i.e., pH and H_2_O_2_) within the biofilm, driving shifts in the local communities [41]. These changes in environmental stimuli will change the community composition of the microbiome, with less fit organisms being outcompeted by microbes better suited to these conditions. Alternative ways fungal-bacterial interactions can affect the community structure include spatial rearrangements. It is now widely accepted that many bacteria adhere to fungal hyphae [42]. This attachment permits redistribution of bacteria within the discrete layers of medically important biofilms. There is also increasing evidence that the presence of fungi in multi-species communities promotes antimicrobial resistance. This reduced susceptibility to antibiotics appears to be mediated via fungal contributions to the extracellular matrix [43]. Therefore, although our current knowledge of the role of the interactions between fungi and bacteria on the microbiota structure and composition are limited, there is precedent that these interactions play an important role and require further investigation.

### 3.2. Fungal–Bacterial Interactions

Fungi and bacteria can interact on multiple levels making polymicrobial interaction studies complex. These interactions can either be agonistic or antagonistic. The most studied fungal-bacterial interactions are those between *C. albicans* and *Pseudomonas aeruginosa* due to their co-habitation of and medical significance in the cystic fibrosis lung, and burns wounds. In this system, these two opportunistic pathogens display an antagonistic relationship. *P. aeruginosa* secretes 3-oxo C12 homoserine lactone to control *C. albicans* morphogenesis, resulting in restricted hyphal growth [44]. However, quorum sensing only inhibits the initiation of hyphal formation, and does not affect extension of pre-existing hyphae [45]. To overcome this, *P. aeruginosa* has also evolved the ability to bind specifically to *C. albicans* hyphae through attachment to carbohydrate components of the fungal cell wall, and induce hyphal death [42]. *P. aeruginosa* also secretes phenazines (i.e., pyrocyanin) that are toxic to fungi [46]. While high concentrations of phenazines kill *C. albicans*, physiological concentrations permit growth on fermentable carbon sources, but restrict hyphal development [47]. The production of fermented by-products further enhances *P. aeruginosa* phenazine production, which promotes the colonisation of *P. aeruginosa* in the lung [48]. Therefore, *P. aeruginosa* appears to have evolved several mechanisms to manipulate *C. albicans* and restrict its growth to yeast. On one hand, this may seem counterproductive, as in other ecosystems bacteria use fungal hyphae to increase their dispersion, suggesting that *P. aeruginosa* could have evolved the ability to attach to *C. albicans* hyphae to aid its dissemination. Conversely, it is possible that yeast cells are better producers of fermented products than hyphae, which is why *P. aeruginosa* invests significant energy into maintaining *C. albicans* in its yeast form to promote its own colonisation in the host. However, *C. albicans* is not a silent partner in this relationship. *C. albicans* secretes its own quorum-sensing molecule, farnesol, which downregulates the expression of *P. aeruginosa* virulence factors through modulation of the Pseudomonas Quinolone System (PQS) system [49]. The roles these interactions play during infection are still unclear, and it is possible that the host environment determines which interactions will prevail. 

The best-documented agonistic fungal-bacterial interactions occur in the oral cavity during the formation of dental plaque. Binding of *Streptococcus gordonii* or *Streptococcus mutans* to *C. albicans* hyphae results in stable biofilm formation around the surface of the tooth. Binding is mediated via the bacterial surface proteins, CshA, SspA and SspB [50], and the fungal adhesin Als3 [51]. Despite colonising the hyphae, *S. gordonii* do not kill the hyphae. Instead, *S. gordonii* promotes *C. albicans* hyphal development through the secretion of the auto inducing peptide AI-2, and through the inhibition of farnesol repression [52]. However, other quorum sensing molecules secreted by *S. gordonii* and *S. mutans* can exert opposing effects on *C. albicans* morphogenesis, with diffusible signal factor (DFS) and competence stimulating peptide (CSP) both inhibiting hyphal formation [53,54]. Therefore, like the interactions between *P. aeruginosa* and *C. albicans*, the outcome of the interaction between *S. gordonii* and *C. albicans* is likely to be controlled by the local environment. 

### 3.3. Fungal–Fungal Interactions

In addition to interacting with bacteria, fungi also interact with one another. For example, *C. glabrata* is able to bind to *C. albicans* hyphae and hitchhike [55] through the host. This attachment promotes invasion of *C. glabrata* into the oral mucosa and may enhance disseminated *C. glabrata* infections. This discovery has cause for concern as *C. glabrata* is inherently resistant the azole class of antifungals [56], the first drug of choice, making disseminated infection hard to treat. 

The quorum-sensing molecule, farnesol, secreted by *C. albicans*, is also able to control the morphology of other fungi inhibiting hyphal growth, conidiation and germination [57,58]. Although the complete mode of action of farnesol is not known, intracellular cAMP levels are reduced upon treatment with exogenous farnesol in many fungi [57], suggesting that inhibition of cAMP signalling pathways is a general trait of farnesol. In addition to inhibiting fungal morphogenesis, farnesol also displays antifungal properties. For example, farnesol induces cell death in multiple fungal species via the generation of reactive oxygen species (ROS) from the mitochondria [59,60,61]. At high concentrations farnesol also induces apoptosis in *C. albicans*, which is dependent on ROS generation [62]. Therefore, *C. albicans* uses farnesol in antagonistic relationships with other fungi to reduce competition in the host, and to control its own growth and morphology by eliciting different responses to specific threshold concentrations of farnesol. 

### 3.4. Host-Fungal Interactions

Alongside the potential for extensive exchanges between different members of the microbial communities and their concomitant impact, these communities will also interact with cells and systems of the host habitat. Whilst a key component of these interactions are those between the fungi and the host innate and adaptive immune system, the specifics of these interactions are covered elsewhere in this special issue. Thus, here, we will focus on the non-immune interactions between host and mycobiome. 

One of the more intriguing phenomena that may result from colonization by the mycobiome is “training” of the innate immune system. Recent studies have indicated that pre-exposure of macrophages to fungal cell wall products (β-glucan) results in epigenetic changes that ultimately lead to a stronger response on infection with live fungi at a later date [63]. Thus, the presence of a mycobiome may result in stronger innate protective responses to all microbes. Whether there is any specificity of this response to key species remains to be determined.

As well as the mycobiome impacting on the host, the host can also have a significant impact on the mycobiome. The presence of microbes and their metabolites (such as short-chain fatty acids) leads to the production of a cocktail antimicrobial peptides (AMPs) that in turn can regulate the species present in the gut. The impact that this circuit can have on the microbiota and health is clearly demonstrated by work investigating the role of NLRP6 in colonic epithelial cells on health [64,65]. This work demonstrates that loss of a detection mechanism can lead to shifts in the microbiota and associated metabolome that can then impact on microbiome composition. Importantly, these shifts are maintained in an otherwise normal genetic background host due to the shift in metabolome. From this, we can see that an ability to model the interactions between host, mycobiome and metabolome will be extremely powerful tool in determining the role of host-mycobiome interactions in both health and disease.

## 4. Modeling of the Mycobiome, Microbiome and Host Interactions

Metagenomic analysis can provide information for the genes and species of the bacteria and potentially fungi, and through using different functional databases such as KEGG, the metabolic functions of these communities can be determined. However, due to the extreme complexity of human microbial ecosystems, multi-omics analyses are incapable of dissecting the overall metabolism of these ecosystems from community-level to individual level and thus elucidating the interactions between microbial species/strains, microbe and host, and other environmental factors. In the study of these complex biological ecosystems, mathematical modeling can provide critical insights that will assist in understanding the underlying mechanisms of these complex systems through the evaluation and testing of different hypothesis. Among these mathematical models, genome-scale metabolic models (GEMs) are perhaps the most important, and have been used to understand the molecular mechanisms of individual organisms in a biological system through the analysis of genotype-phenotype relationships [66]. Tissue/cell specific GEMs have been successfully applied to both human health and disease, to identify novel biomarkers for early diagnosis and efficient treatment of a variety of conditions, such as non-alcoholic fatty liver disease and certain cancer cell-types [67,68]. GEMs have shown their worth and utility in the study of fungi, through prediction of their phenotype in taking up different substrates, the effects of gene knockouts and as a platform for network independent analyses to identify key metabolites and sub-networks [69,70]. Recently, these powerful tools have been applied to the study of microbial communities, such as human gut microbiome [69]. Using GEMs in community metabolic modeling can successfully predict the contribution of individual species and interactions between them to the overall simplified community metabolism and elucidate the interactions between the bacteria [71,72]. Through the generation of comprehensive toolboxes for community modeling, such as CASINO (Community And Systems-level INteractive Optimization), and the use of GEMs for predominant bacteria in human gut, the alteration in the amino acid profile of both feces and serum in response to diet interventions can be simulated and validated [73]. These successful examples of metabolic modeling of human tissue/cell-lines, fungi, and microbiome communities pave the way for the application of these methods on mycobiome research, enabling us to better understand the interactions between fungi and bacteria, other fungi and their host habitat; this allows us to elucidate their role in different diseases, alongside their overall contributions in human host-microbial metabolism. 

## 5. Conclusions

As we develop an improved understanding of the pivotal role played by microbial communities in health and disease, we also increase our appreciation for the key role played by fungal communities in these situations. These fungal communities unsurprisingly show significant variation between different body habitats and with changes in disease status. We are beginning to grasp the significant role that these variations play in host homeostatic responses and pathologies, although our understanding here is still very much in its infancy. As we develop an increasing understanding of how factors such as host and microbial responses impact on the mycobiome and, likewise, how the mycobiome affects other microbial communities and the host, so we will improve our ability to predict the significance of changes in the mycobiome on host status. As we move forward, the importance and significance of advanced in silico modeling techniques (such as GEMs) associated with systems biology will be of ever-increasing importance, enabling us to create even more complex predictions of the role of different species, cell types and metabolites, with the ultimate goal of being able to determine specific, personalized interventions that improve the health of an individual.
